# Is red blood cell distribution width a marker of severity in patients discharged from the ICU?

**DOI:** 10.1186/s40560-019-0364-6

**Published:** 2019-01-29

**Authors:** Tiago Antonio Tonietto, Marcio Manozzo Boniatti, Thiago Costa Lisboa, Marina Verçoza Viana, Gustavo Adolpho Moreira Faulhaber

**Affiliations:** 1 Department of Critical Care Medicine, Hospital Nossa Senhora da Conceição, 596 Francisco Trein Ave, Porto Alegre, RS 91350-200 Brazil; 2 Department of Critical Care Medicine, Hospital de Clínicas de Porto Alegre, 2350 Ramiro Barcelos Street, Porto Alegre, RS 90035-903 Brazil; 3 Department of Internal Medicine, School of Medicine, Universidade Federal do Rio Grande do Sul, 721 Jeronimo de Ornelas Ave, Porto Alegre, RS 90040-341 Brazil

**Keywords:** Red blood cell distribution width, RDW, Intensive care unit, Mortality

## Abstract

We have read the study about the association between high red blood cell distribution width and higher ward mortality after intensive care unit discharge. The study increases the evidence that RDW may be a marker of severity for patients discharged from the ICU. However, in this letter, we comment on issues that need further discussion.

Dear Editor,

We read with interest the retrospective study by Fernandez and colleagues entitled “High red blood cell distribution width as a marker of mortality after ICU discharge: a cohort study” [1]. The authors evaluated the association between the red blood cell distribution width (RDW) with ward survival after intensive care unit (ICU) discharge and whether the RDW could improve the accuracy of the Sabadell score, a subjective tool designed to stratify patients according to ward mortality after ICU discharge. They showed that high RDW (defined as greater than 14.5%) was associated with increased ward mortality (multivariable OR 2.8 [1.7–4.6]) and longer ward stay. Additionally, high RDW identified patients with higher ward mortality at each level of prognosis depicted by the Sabadell score, except for score 3 group. This interesting study increases the evidence that the RDW may be a marker of severity for patients discharged from the ICU. However, there are issues that need further discussion.

Firstly, in the methods section, it is stated that routine laboratory was requested by the attending physician as clinically required and the authors used the last recorded RDW before discharge to the ward. However, in the results, there should have been a description of the mean time between the RDW collection and the moment of ICU discharge. This is specially important because the median ICU length of stay was < 3.5 days and the authors did not adjust their results for the RDW collected in the ICU admission. Thus, the ward mortality could be related to high RDW at ICU admission and not at ICU discharge. The worst outcomes associated with the former have already been demonstrated in other studies [2].

Additionally, it would be interesting if the authors had reported the variation between the RDW collected at ICU discharge and ICU admission. Few studies have assessed the kinetics of the RDW and its prognostic value throughout hospitalization [3, 4]. Our group described that patients readmitted to the ICU or who died in the ward had a significantly more pronounced increase in RDW during their ICU stay than other patients (median = 0.5% and 0.2%, respectively; *p* = 0.02) [5].

Lastly, we have also demonstrated that high RDW is a marker of severity in patients discharged from the ICU to the wards [5]. We illustrated that patients with elevated RDW at ICU discharge had a significantly higher risk of ICU readmission or unexpected death in ward than patients with normal RDW (HR 1.901; 95% CI 1.357–2.662), even after adjusting for the RDW at ICU admission and several known risk factors associated with this outcome. Furthermore, elevated RDW at discharge from the ICU was also associated with hospital mortality (HR 1.716; 95% CI 1.141–2.580).

Finally, practical implications of these findings include developing clinical prediction rules that consider the RDW as a relevant variable at ICU discharge. The impact of recognition of the RDW as a risk factor, leading to closer monitoring of these patients in wards, will depend on further studies assessing specific interventions.

## Abbreviations

HR: Hazard ratio
ICU: Intensive care unit
OR: Odds ratio
RDW: Red blood cell distribution width
